# Maternal tributyrin supplementation in late pregnancy and lactation improves offspring immunity, gut microbiota, and diarrhea rate in a sow model

**DOI:** 10.3389/fmicb.2023.1142174

**Published:** 2023-04-12

**Authors:** Yan Lin, Dan Li, Zhao Ma, Lianqiang Che, Bin Feng, Zhengfeng Fang, Shengyu Xu, Yong Zhuo, Jian Li, Lun Hua, De Wu, Junjie Zhang, Yuanxiao Wang

**Affiliations:** ^1^Key Laboratory of Animal Disease-Resistance Nutrition and Feed Science, Institute of Animal Nutrition, Sichuan Agricultural University, Chengdu, Sichuan, China; ^2^Key Laboratory of Animal Disease-Resistance Nutrition, Ministry of Education, Chengdu, Sichuan, China; ^3^College of Life Science, Sichuan Agricultural University, Ya’an, Sichuan, China; ^4^Perstorp (Shanghai) Chemical Trading Co., Ltd., Shanghai, China

**Keywords:** sows, piglets, tributyrin, fecal microbial, diarrhea rate

## Abstract

**Introduction:**

Several studies have evaluated the effects of tributyrin on sow reproductive performance; however, none of these studies have investigated the effects of tributyrin on sow gut microbiota and its potential interactions with immune systems and milk composition. Therefore, we speculated that tributyrin, the combination of butyrate and mono-butyrin without odor, would reach the hindgut and affect the intestinal microbiota composition and play a better role in regulating sow reproductive performance, gut flora, and health.

**Methods:**

Thirty sows (Landrace × Yorkshire) were randomly divided into two groups: the control group (CON) and the tributyrin group (TB), which received basal diet supplemented with 0.05% tributyrin. The experimental period lasted for 35 days from late pregnancy to lactation.

**Results:**

The results showed that TB supplementation significantly shortened the total parturition time and reduced the diarrhea rate in suckling piglets. On day 20 of lactation, the milk fat and protein levels increased by 9 and 4%, respectively. TB supplementation significantly improved the digestibility of dry material, gross energy, and crude fat in the sow diet, but had no significant effect on crude protein digestibility. Furthermore, TB supplementation increased the levels of IL-10, IL-6, and IgA in the blood of weaned piglets, but had no effect on maternal immunity. Analysis of the fecal microbial composition revealed that the addition of TB during late gestation and lactation increased the microbiota diversity in sows and piglets. At the phylum level, sows in the TB group had a slight increase in the relative abundance of Bacteroidota and Spirochaetota and a decrease in Firmicutes. At the order level, the relative abundance of Lactobacillales was increased in piglets and sows, and the TB group showed increased relative abundance of Enterobacterales and significantly decreased relative abundance of Oscillospirales in piglets. At family level, the relative abundance of Lactobacillaceae, Oscillospiraceae, and Christensenellaceae increased in sows, and the relative abundance of Enterobacteriaceae and Lactobacillaceae increased in piglets. At genus level, the relative abundance of *Lactobacillus* increased in sows and piglets, but the relative abundance of *Subdoligranulum* and *Eubacterium_fissicatena*_group decreased in piglets in the TB group.

**Discussion:**

In conclusion, tributyrin supplementation shortened the farrowing duration and reduced the diarrhea rate of piglets by improving the inflammatory response and composition of gut microbiota in piglets and sows.

## 1. Introduction

Butyrate, a short-chain fatty acid, is a key gut microbial metabolite that mediates the effect of the gut microbiome on the immune system and plays a key role in maintaining intestinal immune homeostasis (Siddiqui and Cresci, 2021). It is often used as a feed additive because it helps improve the health of the intestinal flora and maintain intestinal immune homeostasis. Research has shown that supplementation of pregnant sows with 0.3% dietary butyrate increases average daily weight gain (Lu et al., 2012), changes colostrum composition, and improves the growth rate of piglets (He et al., 2016). In addition, after butyrate supplementation, the immune function of newborn piglets is changed by reducing the production of TNF-α and increasing the concentration of IgA in colostrum, thereby improving their growth rate (He et al., 2016) and greatly reducing their pre-weaning mortality rate (Wang et al., 2019a,b; Wang H. et al., 2019). However, butyrate is also volatile and corrosive. In practice, supplementary butyrate is mostly added as sodium butyrate, and it is easily absorbed by the upper digestive tract (Moquet et al., 2016). Moreover, its smell is unbearable (Mallo et al., 2012), leading to a decrease in feed intake (Biagi et al., 2007); therefore, its direct use in animal production is very difficult.

Tributyrin is a valid alternative to butyrate, as one molecule of tributyrin releases three molecules of butyrate directly in the small intestine (Sotira et al., 2020) and can reach the hindgut more effectively as compared to butyrate (Augustin et al., 2011). Thus, it may affect gut microbiota composition and health. Tributyrin does not decompose in gastric juice and is slowly released into butyrate and glycerol under the action of pancreatic lipase (Miyoshi et al., 2011). Tributyrin promotes intestinal mucosal growth (Wang et al., 2019a,b; Wang H. et al., 2019), regulates intestinal microbial community changes (Gong et al., 2021; Miragoli et al., 2021), increases protein absorption, utilization, and synthesis (Sotira et al., 2020), regulates piglet metabolism, improves piglet growth performance, significantly improves average daily weight gain, and reduces stool score of weaned piglets (Wang et al., 2019b). The addition of tributyrin at 2 g/kg in the diet prevents growth retardation by stimulating the appetite of weaned pigs, regulates inflammatory cytokine production *in vivo* to prevent fatal infection in weaned pigs, and improves the growth performance of piglets (Gu et al., 2017). However, only a few studies have investigated the effects of tributyrin supplementation on sow reproductive performance and health. The addition of 0.1% tributyrin also improves the growth and intestinal digestion and barrier function of intrauterine growth-restricted (IUGR) piglets during lactation (Dong et al., 2016). The addition of 1,000 mg/kg tributyrin to broiler diets improves their reproductive performance, antioxidant capacity, and ovarian function (Wang J. et al., 2021; Wang Y. et al., 2021). In addition, diet supplemented at 250 mg/kg tributyrin improves growth performance by regulating blood biochemical indicators and cecal microbial community composition in broilers (Gong et al., 2021).

Several studies have evaluated the effects of tributyrin on sow reproductive performance; however, none of these studies have investigated the effects of tributyrin on sow gut microbiota and its potential interactions with immune systems and milk composition. Therefore, we speculated that tributyrin, the combination of butyrate and mono-butyrin without odor, would reach the hindgut and affect the intestinal microbiota composition and play a better role in regulating sow reproductive performance, gut flora, and health.

## 2. Materials and methods

### 2.1. Animal and dietary management

This study was conducted on a commercial pig farm. The experiments followed the actual law of animal protection, was approved by the Institutional Animal Care and Use Committee of Sichuan Agricultural University (SCAUAC202108-3) and was conducted in accordance with the Guide for the Care and Use of Laboratory Animals of the National Research Council.

Thirty Landrace × Yorkshire sows with similar parity and backfat thickness (15.59 ± 2.63) were selected and randomly divided into two groups (*n* = 15 each): the control group (CON) was fed a basic diet and the tributyrin group (TB) was fed a basic diet supplemented with 500 g/t tributyrin (ProPhorce™ SR 130, provided by Perstorp Shanghai Company, butyrate content ≥51.4%). The diet was designed according to the experimental design and nutritional needs of the NRC (2012) pigs. Supplementary Table 1 lists the nutritional parameters of the gestation and lactation diets.

During the experiments, all sows were fed twice daily, at 8:30 and 14:30, with free access to water. From day 90 to day 110 of gestation, sows were fed 2.8 kg/day. The feed allowance was reduced gradually to 2.0 kg/day in the 3 days immediately before parturition. After parturition, sows were fed 2 kg/day on lactation on day 1, then increased by 1.0 kg/day until the animal fed freely. After delivery, piglets were weighed one by one, and the number of piglets per sow was adjusted to 10 ± 1 within 24 h postpartum. All the piglets drank freely and received no additional feed. All piglets were weaned on day 21 of lactation and the mammalian temperature was maintained at 20–25°C.

### 2.2. Data measurement and collection

Body weight and backfat thickness were measured after delivery and weaning for each sow. The back fat thickness of the sows was measured at 6.5 cm to the level of the last rib from the dorsal mid-line using an ultrasonic device (Renco Lean-Meatier; Renco Corporation, Minneapolis, MN, United States). The total litter size, number of piglets born alive, weight after birth, and weight on day 21 were recorded, and the weaning survival rate, daily weight gain, and litter weight gain were calculated. During the entire trial period, the fecal morphology of the sows was checked and recorded at a fixed time every day, and the degree of constipation was scored according to Oliviero et al. (2010): 0 (soft), 1 (particle size but soft), 2 (normal feces), 3 (moderate constipation), and 4 (severe constipation), and the constipation rate was calculated repeatedly. During lactation, piglets scored stool morphology according to a 4-point system, where a diarrhea score ≥ 2 was judged as diarrhea (Long et al., 2021).

### 2.3. Sample collection

Feed samples were collected by quartering and were hermetically stored at −20°C. On day 14 of lactation, fecal samples of sows were collected for five consecutive days and stored at −20°C for nutrient digestibility by endogenous indicator method. After collection, the fecal samples from each pig were mixed well, and approximately 200 g of the samples were collected by quartering and hermetically stored at −20°C. The fecal samples of sows and piglets were collected on day 21 of lactation for microbial analysis. Twelve litters were selected for each treatment, and a piglet close to the average weight were selected for each litter. The feces of sows and piglets in all treatment groups were collected by rectal massage method and stored hermetically at −20°C. On day 0 and 20 after parturition, blood samples (10 mL) were collected by ear venipuncture. On day 21 of lactation, blood from lactating piglets (5 mL) was collected from the anterior vena cava. All blood samples were centrifuged at 3,000× *g* at 4°C for 10 min, and serum was separated and immediately stored at −20°C for later analysis. Colostrum samples were collected from the fourth or fifth pair (any nipple or more nipples) breast after the birth of the fifth piglet. On day 20 of lactation, milk samples were collected from sows by injecting 1.0 mg of oxytocin into the auricular vein and stored at −20°C for later analysis.

### 2.4. Determination of nutrient digestibility

Dry matter (DM), crude protein (CP), crude fat (*CF*), acid detergent fiber (ADF), and neutral detergent fiber (NDF) in the feed and fecal samples were measured according to AOAC (2012), and the apparent total tract digestibility (ATTD) of each nutrient was calculated.

ATTD=[1−(b÷n)×(c÷d)]×100%

Where: *n* is the content of a nutrient in the diet; *b* is the content of a nutrient in the fecal sample; *c* is the content of the indicator in the test diet; and *d* is the content of the indicator in the fecal sample Acid-insoluble ash was determined with reference to GB/T 23742–2009.

### 2.5. Plasma hormone and biochemical analyses

Plasma metabolites from sows (glucose, total cholesterol, triglycerides, and urea) were determined using HITACHI 3100 Automatic Analyzer (Hitachi High-Tech Science Systems Inc., Tokyo, Japan). Plasma concentrations of reproduction-related regulatory hormones (insulin, leptin, prolactin, and GLP-1) in sows were determined using commercial ELISA kits (Nanjing Jiancheng Bioengineering Institute, Nanjing, China), according to the manufacturer’s protocol.

### 2.6. Chemical composition of colostrum and milk analysis

The concentrations of fat, protein, lactose, total solids, and urinary nitrogen in colostrum and milk were determined using an automatic milk composition analyzer (Foss MilkoScan FT+, Fossomatic FC). The concentrations of interleukin-A (IgA) and interleukin-A (IgM) were determined by the commercial ELISA kits (Nanjing Jiancheng Bioengineering Institute, Nanjing, China).

### 2.7. Determination of immunoglobulin and cytokine levels

Interleukin-1β (IL-1β), interleukin-6 (IL-6), interleukin-G (IgG), IgA, interleukin-M (IgM), Interleukin-2 (IL-2), and Interleukin-10 (IL-10) levels in the blood of sows and piglets were determined using commercial ELISA kits (Nanjing Jiancheng Bioengineering Institute, Nanjing, China) and an Absorbance Microplate Reader (SpectraMax190).

### 2.8. Fecal microbial analysis

Fecal samples were evaluated using 16S rDNA sequencing to determine the microbial composition of feces collected from sows and piglets. Bacterial DNA was isolated from fecal samples using an E.Z.N.A.^®^ Soil DNA Kit (Omega Bio-tek, Norcross, Georgia, United States). Using this DNA as a template, the V3-V4 hypervariable regions of the bacterial 16S rRNA gene were amplified using primers F338 (5′-ACTCCTACGGGAGGCAGCAG-3′) and R806 (5′-GGACTACHVGGGTWTCTAAT-3′). An AxyPrep DNA Gel Extraction Kit was used to purify the amplicons. Purified amplicons were paired-end sequenced on an Illumina MiSeq PE300 platform/NovaSeq PE250 platform (Illumina, San Diego, California, United States). Operational taxonomic units (OTUs) with a 97% similarity cut-off were clustered, and chimeric sequences were identified and removed. The taxonomy of the representative sequence of each OTU was analyzed (Wang et al., 2007) with a confidence threshold of 0.7.

### 2.9. Statistical analysis

One-way ANOVA was performed using SAS 9.0 for sow reproductive performance, nutrient digestibility, milk composition, and blood parameters, and all data are expressed as mean ± SEM. A *p* < 0.05 was considered significant for all analyses, whereas a 0.05 < *p* < 0.10 was considered a tendency.

## 3. Results

### 3.1. Effects of tributyrin on the reproductive performance of sows

As shown in Table 1, the TB group had a shortened total length of delivery (*p* < 0.05) but showed no significant difference in litter size, live litter size, primary weight of piglets, or constipation score (*p* > 0.05). However, the constipation rate was reduced by 31% (*p* > 0.05).

**Table 1:** Effects of tributyrin on delivery performance and fecal score of sows.

Item	CON	TB
Labor length, (min)	229.27 ± 15.43^b^	177.01 ± 12.56^a^
Interdelivery interval, (min)	22.49 ± 2.43^b^	14.26 ± 2.74^a^
Number of total born	11.69 ± 1.04	12.19 ± 0.58
Number of liveborn	11.00 ± 0.93	11.50 ± 0.87
Litter weight of piglets, (kg)	15.18 ± 1.36	14.76 ± 0.94
Weight of piglets, (kg)	1.41 ± 0.34	1.33 ± 0.26
Constipation rate, (%)	12.20 ± 2.32	8.33 ± 1.07
Constipation scoring	1.66 ± 0.14	1.38 ± 0.22

CON, basal diet; TB, basal diet + 500 g/t tributyrin. ^a,b^Values in the same row with different small letter superscripts mean significant difference (*p* < 0.05). Data are presented as mean ± SEM (*n* = 15).

### 3.2. Effects of tributyrin on feed intake and backfat change in sows

As shown in Table 2, the TB group had no significant difference on feed intake or backfat loss (*p* > 0.05).

**Table 2:** Effects of tributyrin on feed intake and backfat thickness during lactation in sows.

Items	CON	TB
Feed intake, kg		
Day 0–7	4.77 ± 0.34	4.56 ± 0.51
Day 8–14	6.17 ± 0.46	6.10 ± 0.44
Day 14–21	6.27 ± 0.64	6.22 ± 0.59
Day 0–21	5.790 ± 0.55	5.682 ± 0.43
Backfat thickness (mm)		
BF on the day of deliver	15.27 ± 3.21	14.93 ± 2.04
BF on weaning	13.50 ± 1.45	13.52 ± 2.18
BF loss during lactation	1.77 ± 0.48	1.43 ± 0.55

CON, basal diet; TB, basal diet + 500 g/t tributyrin. BF, backfat thickness. Data are presented as mean ± SEM (*n* = 15).

### 3.3. Effects of tributyrin on the growth performance of weaning piglets

As shown in Table 3, no significant difference was noted in weaning survival rate, litter weight, or individual body weight, but the TB group had increased litter weight of piglets by 3–5%, weight of piglets by 200 g (*p* > 0.05), and significantly reduced rate of diarrhea in lactating piglets (*p* < 0.05).

**Table 3:** Effects of tributyrin on growth performance of weaning piglets.

Items	CON	TB
Number of piglet per litters	10.21 ± 0.55	11.67 ± 0.46
Survival rate, (%)	91.96 ± 2.15	92.87 ± ±1.88
Litter weight, (kg)	59.71 ± 2.48	62.85 ± 3.01
BW, (kg)	6.15 ± 0.26	6.35 ± 0.35
ADG, (g/d)	225.77 ± 16.78	238.72 ± 22.17
Diarrhea score	0.67 ± 0.15^b^	0.33 ± 0.09^a^
ID, (%)	0.92 ± 0.24^b^	0.20 ± 0.08^a^

CON, basal diet; TB, basal diet + 500 g/t tributyrin. ^a,b^Values in the same row with different small letter superscripts mean significant difference (*P* < 0.05). BW, body weight; ADG, average daily gain; ID, Incidence of diarrhea. Data are presented as mean ± SEM (*n* = 15).

### 3.4. Effects of tributyrin on the composition of colostrum and normal milk in sows

As shown in Table 4, no significant difference was noted in the composition of colostrum or normal milk in sows, but the IgA in colostrum increased by 11.8%. In contrast, milk fat, milk protein, and total solids in milk on day 20 of lactation were, respectively, increased by 9% (*p* = 0.089), 4%, and 7.6%.

**Table 4:** Effects of tributyrin on composition of colostrum and regular milk in sows.

Items	CON	TB
Colostrum		
Fat, (%)	7.25 ± 0.49	7.51 ± 0.37
Protein, (%)	14.55 ± 1.36	15.17 ± 0.98
Lactose, (%)	2.75 ± 0.29	2.77 ± 0.30
Total solids, (%)	28.56 ± 2.07	29.49 ± 2.19
Urinary nitrogen, (mg/dL)	82.28 ± 7.46	86.36 ± 6.28
IgA, (mg/mL)	4.06 ± 1.06	4.54 ± 1.48
IgM, (mg/mL)	0.74 ± 0.05	0.72 ± 0.06
Milk at day 20 of lactation		
Fat, (%)	6.65 ± 0.45	7.32 ± 0.36
Protein, (%)	5.02 ± 0.38	5.23 ± 0.25
Lactose, (%)	5.52 ± 0.56	5.57 ± 0.32
Total solids, (%)	19.32 ± 1.36	20.78 ± 2.02
Urinary nitrogen, (mg/dL)	72.64 ± 4.48	73.96 ± 5.09

CON, basal diet; TB, basal diet + 500 g/t tributyrin. Data are presented as mean ± SEM (*n* = 15).

### 3.5. Effects of tributyrin on blood biochemical markers in sows and piglets

As shown in Table 5, the addition of tributyrin had no significant effect in the blood biochemical indexes of sows but had a tendency to increase urinary nitrogen in the blood on day 20 of lactation (*p* = 0.092). In addition, blood triglycerides of piglets increased by 16% in the TB group.

**Table 5:** Effects of tributyrin on blood biochemical indexes of sows.

Items	CON	TB
Glucose, (mmol/L)		
Sows on day 0 of lactation	3.36 ± 0.32	3.13 ± 0.35
Sows on day 20 of lactation	5.20 ± 0.41	5.24 ± 0.48
Weaned piglets	7.21 ± 0.15	7.22 ± 0.16
Urinary nitrogen, (mmol/L)		
Sows on day 0 of lactation	3.60 ± 0.43	3.92 ± 0.29
Sows on day 20 of lactation	5.23 ± 0.22	5.96 ± 0.34
Weaned piglets	2.54 ± 0.33	2.91 ± 0.41
Total cholesterol, (mmol/L)		
Sows on day 0 of lactation	1.74 ± 0.06	1.43 ± 0.14
Sows on day 20 of lactation	2.81 ± 0.55	2.41 ± 0.48
Weaned piglets	6.11 ± 0.66	6.03 ± 0.57
Triglycerides, (mmol/L)		
Sows on day 0 of lactation	0.30 ± 0.05	0.26 ± 0.04
Sows on day 20 of lactation	0.22 ± 0.04	0.26 ± 0.05
Weaned piglets	1.28 ± 0.09	1.48 ± 0.14

CON, basal diet; TB, basal diet + 500 g/t tributyrin. Data are presented as mean ± SEM (*n* = 8).

### 3.6. Effects of tributyrin on nutrient digestibility in lactating sows

As shown in Table 6, the addition of tributyrin significantly improved the digestibility of dry material, energy (*p* < 0.01), and crude fat (*p* < 0.05) in the sow diet, but had no significant effect on crude protein digestibility.

**Table 6:** Effects of tributyrin on dietary nutrient digestibility in sows.

Digestibility, (%)	CON	TB
Dry matter	94.10 ± 0.38 ^A^	94.94 ± 0.27 ^B^
Crude protein	85.37 ± 1.31	86.45 ± 0.90
Gross energy	80.48 ± 0.70^A^	81.58 ± 0.39^B^
Crude fat	74.75 ± 1.32^a^	77.41 ± 1.08^b^

CON, basal diet; TB, basal diet + 500 g/t tributyrin. ^a,b^Values in the same row with different small letter superscripts mean significant difference (*P* < 0.05). ^A,B^Values mean highly significant difference(*P* < 0.01). Data are presented as mean ± SEM (*n* = 8).

### 3.7. Effects of tributyrin on hormone secretion in lactating sows

As shown in Table 7, the addition of tributyrin had no significant effect on the secretion of GLP-1, INS, or LEP in the blood on days 0 and 20 of lactation, but had a tendency to improved PRL in the blood on day 0 of lactation (*p* = 0.058) and decreased INS in the blood on day 20 of lactation (*p* = 0.086).

**Table 7:** Effects of tributyrin on blood hormone secretion in sows.

Items	CON	TB
Day 0 of lactation		
GLP-1, (pmol/L)	5.26 ± 0.89	6.89 ± 1.89
INS, (mIU/L)	69.39 ± 11.90	69.49 ± 10.08
LEP, (ng/mL)	12.26 ± 4.43	17.48 ± 4.25
PRL (ng/ mL)	1961.62 ± 158.23	2677.85 ± 117.79
Day 20 of lactation		
GLP-1, (pmol/L)	6.17 ± 0.88	8.16 ± 1.49
INS, (mIU/L)	71.81 ± 6.26	59.87 ± 2.10
LEP, (ng/mL)	19.70 ± 4.69	14.07 ± 3.96
PRL, (ng/mL)	2892.15 ± 219.85	3270.85 ± 195.71

CON, basal diet; TB, basal diet + 500 g/t tributyrin. GLP-1, glucagon-like peptide-1; INS, Insulin; LEP, Leptin; PRL, prolactin. Data are presented as mean ± SEM (*n* = 8).

### 3.8. Effects of tributyrin on blood immunoglobulin and cytokine secretion

As shown in Table 8, the addition of tributyrin did not have significant effects on IL-6, IL-10, IgA, IgG or IgM concentrations in sow serum on days 0 and 20 of lactation. Interestingly, dietary tributyrin supplementation in sows increased the levels of IL-6 and IgA (*p* < 0.05) in the blood of 21-day-old lactating piglets with a tendency to increase the concentration of IL-10 (*p* < 0.10).

**Table 8:** Effects of tributyrin on blood hormone secretion in sows and weaned piglets.

Items	CON	TB
Sows on day 0 of lactation		
IL-6, (ng/L)	61.94 ± 6.96	64.30 ± 7.89
IL-10, (ng/L)	124.73 ± 13.26	132.45 ± 11.48
IgA, (mg/mL)	1.21 ± 0.14	1.20 ± 0.18
IgG, (mg/mL)	4.22 ± 0.54	4.47 ± 0.38
IgM, (mg/mL)	0.695 ± 0.06	0.649 ± 0.05
Sows on day 20 of lactation		
IL-6, (ng/L)	60.69 ± 2.08	56.54 ± 2.87
IL-10, (ng/L)	124.19 ± 8.99	117.04 ± 6.11
IgA, (mg/mL)	1.17 ± 0.20	1.21 ± 0.23
IgG, (mg/mL)	4.97 ± 0.41	4.59 ± 0.48
IgM, (mg/mL)	0.404 ± 0.15	0.399 ± 0.10
Piglets on day 21 of lactation		
IL-1β, (ng/L)	68.58 ± 8.95	68.59 ± 7.65
IL-6, (ng/L)	57.85 ± 5.20^a^	71.17 ± 7.18^b^
IL-10, (ng/L)	149.14 ± 10.32	171.61 ± 7.75
IgA, (mg/mL)	0.88 ± 0.11^a^	1.17 ± 0.05^b^
IgG, (mg/mL)	5.04 ± 0.27	4.72 ± 0.40

CON, basal diet; TB, basal diet + 500 g/t tributyrin. ^a,b^Values in the same row with different small letter superscripts mean significant difference (*P* < 0.05). Data are presented as mean ± SEM (sows, *n* = 8; piglets, *n* = 12).

### 3.9. Effects of tributyrin on the fecal microbial composition of sows and piglets

**Figure 1:**
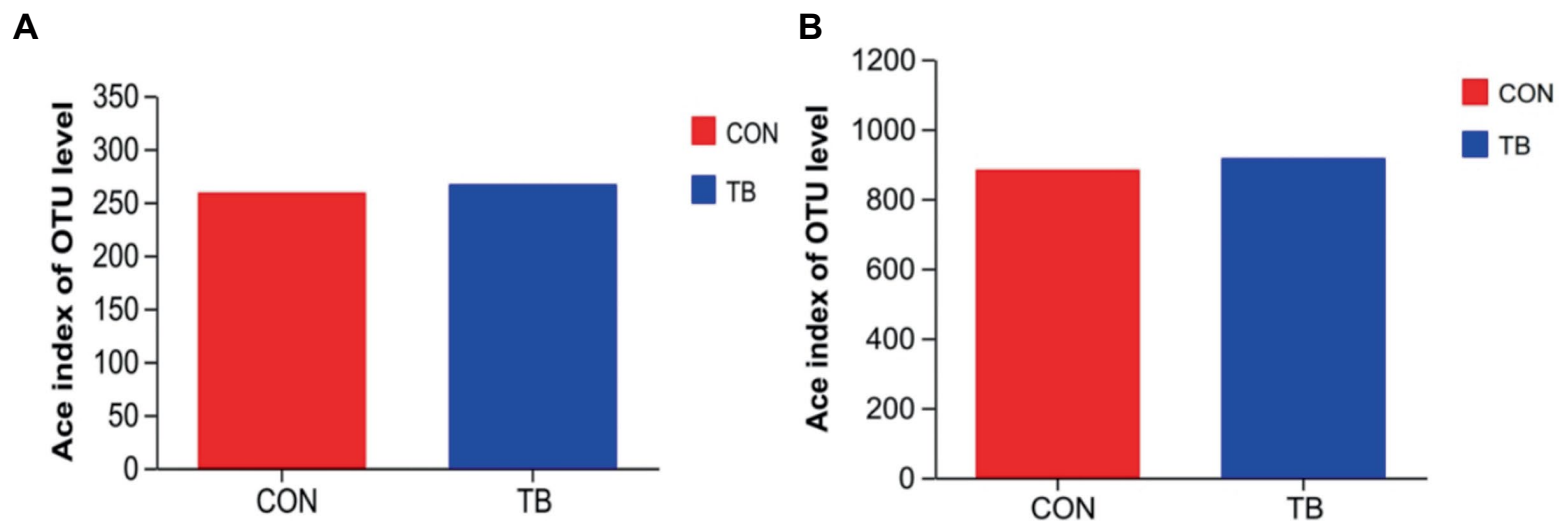
Effects of tributyrin on fecal microorganisms of sows **(A)** and piglets **(B)**. CON, basal diet; TB, basal diet +500 g/t tributyrin.

As shown in Figure 1, there is not statistically significant in the microbiota diversity of sows (Figure 1A) and piglets (Figure 1B).

**Figure 2:**
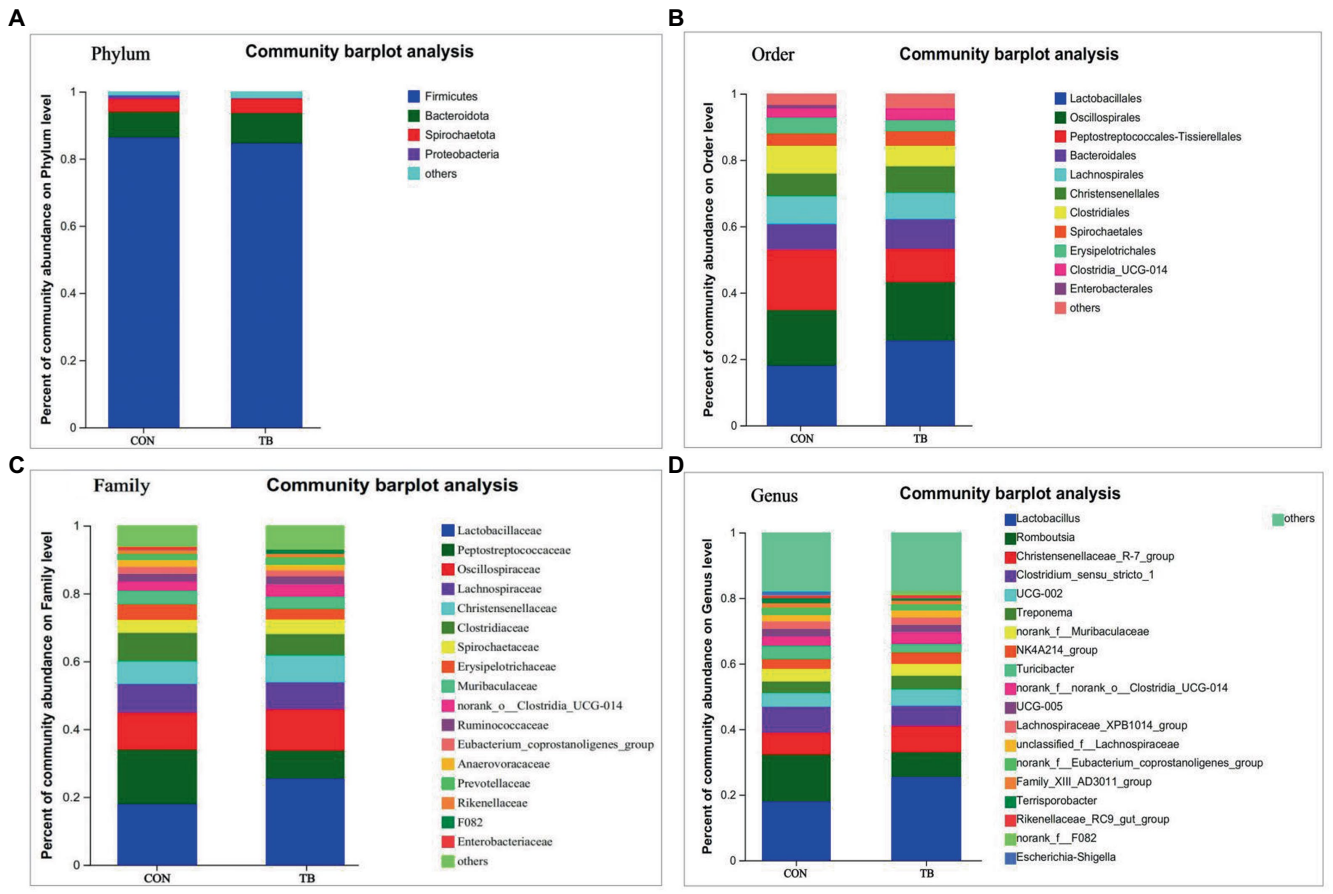
Effects of tributyrin on fecal microbial composition of sows. The overall composition of the sow fecal microflora at the phylum **(A)**, order **(B)**, family **(C)**, and genus **(D)** level is indicated in the bar chart. Each bar graph represents the average relative abundance of each bacterial taxon in a set. The abscissa/ordinate shows the sample name, the ordinate/abscissa shows the proportion of the species in the sample, columns of different colors represent different species, and the length of the columns represents the proportion of the species.

Compared with the control, the microbiota of the fecal samples in the TB group showed a slight increase in the relative abundance of Bacteroidota and Spirochaetota, and a decrease in Firmicutes (Figure 2A). As shown in Figure 2B, at the order level, the relative abundance of Lactobacillales was increased in the TB group; there was an increasing trend for the TB group in the relative abundance of Oscillospirales but significantly reduced the relative abundance of Peptostreptococcales*-*Tissierellales (*p* < 0.01). As shown in Figure 2C, at the family level, the TB group increased the relative abundance of Lactobacillaceae, Oscillospiraceae, and Christensenellaceae, and showed a significantly reducing in that of Peptostreptococcaceae (*p* < 0.01) and Clostridiacea. As shown in Figure 2D, TB supplementation increased the relative abundance of *Lactobacillus*, significantly reduced the relative abundance of *Romboutsia* (*p* < 0.01). Therefore, the above results indicate that the gut microbiota composition of sows was profoundly altered during late pregnancy and lactation.

**Figure 3:**
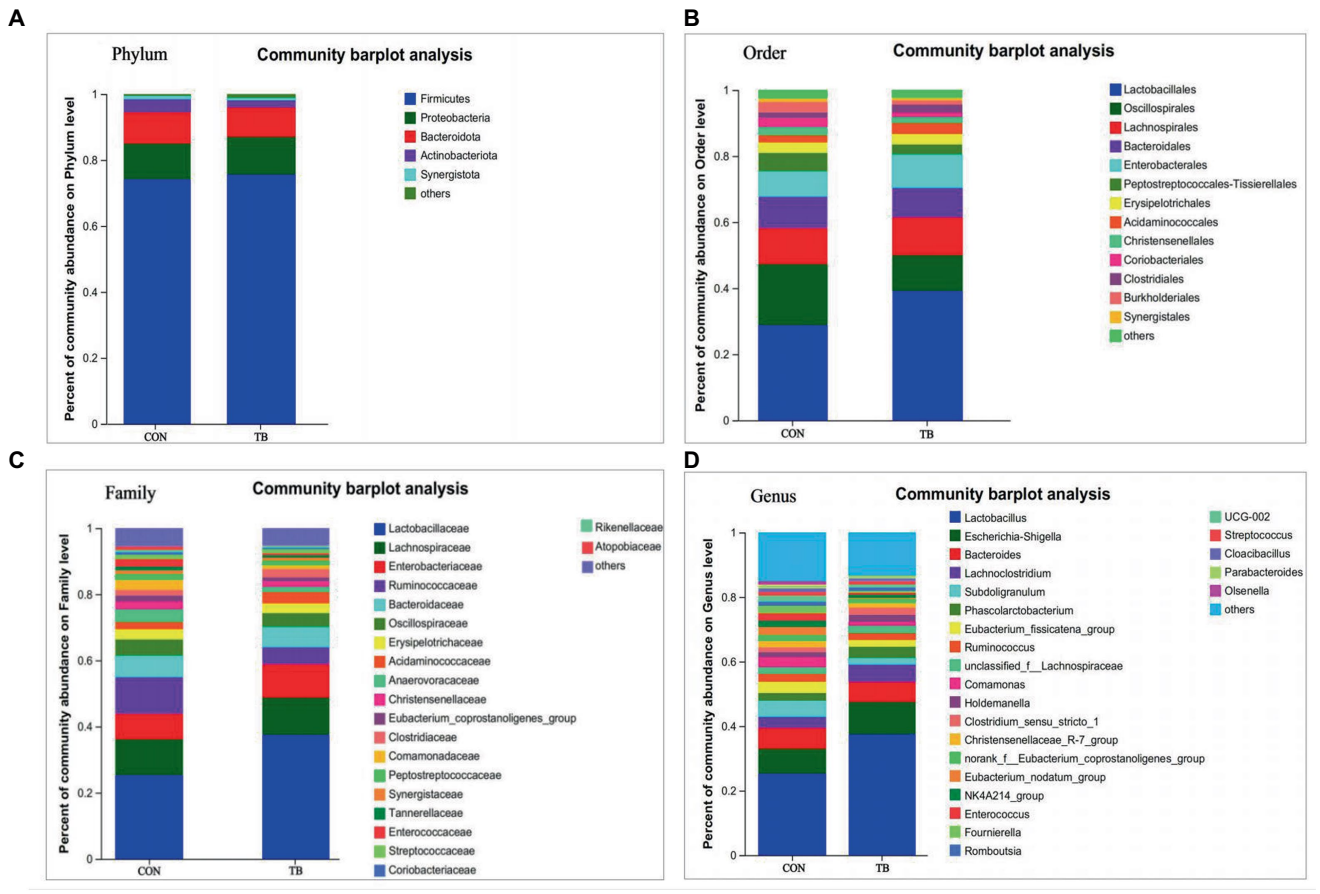
Effects of tributyrin on the fecal microbial composition of piglets. The overall composition of fecal microbial flora in piglets at phylum **(A)**, order **(B)**, family **(C)**, and genus **(D)** level is indicated in the bar charts. Each bar graph represents the average relative abundance of each bacterial taxon in a set. The abscissa/ordinate shows the sample name, the ordinate/abscissa shows the proportion of the species in the sample, columns of different colors represent different species, and the length of the columns represents the proportion of the species.

Figure 3 shows that dietary tributyrin in late pregnancy and lactation had a profound impact on the intestinal microbial composition of offspring piglets. As shown in Figure 3A, the relative abundance of Proteobacteria in the TB group tended to increase at the phylum level. As shown in Figure 3B, at the order level, the TB group increased the relative abundance of Lactobacillales and Enterobacterales and significantly decreased the relative abundance of Oscillospirales (*p* = 0.034). Similarly, the relative abundance of Enterobacteriaceae was also increased in the TB group (Figure 3C), and the TB group increased the relative abundance of Lactobacillaceae, significantly enduce the relative abundance of Ruminococcaceae (*p* = 0.032). As shown in Figure 3D, at the genus level, the TB group increased the relative abundance of *Lactobacillus, Escherichia-Shigella*, and *Lachnoclostridium* and decreased the relative abundance of *Subdoligranulum* and *Eubacterium_fissicatena_group*. Therefore, the above results indicate that the gut microbiota composition of piglets was profoundly altered during lactation.

**Figure 4:**
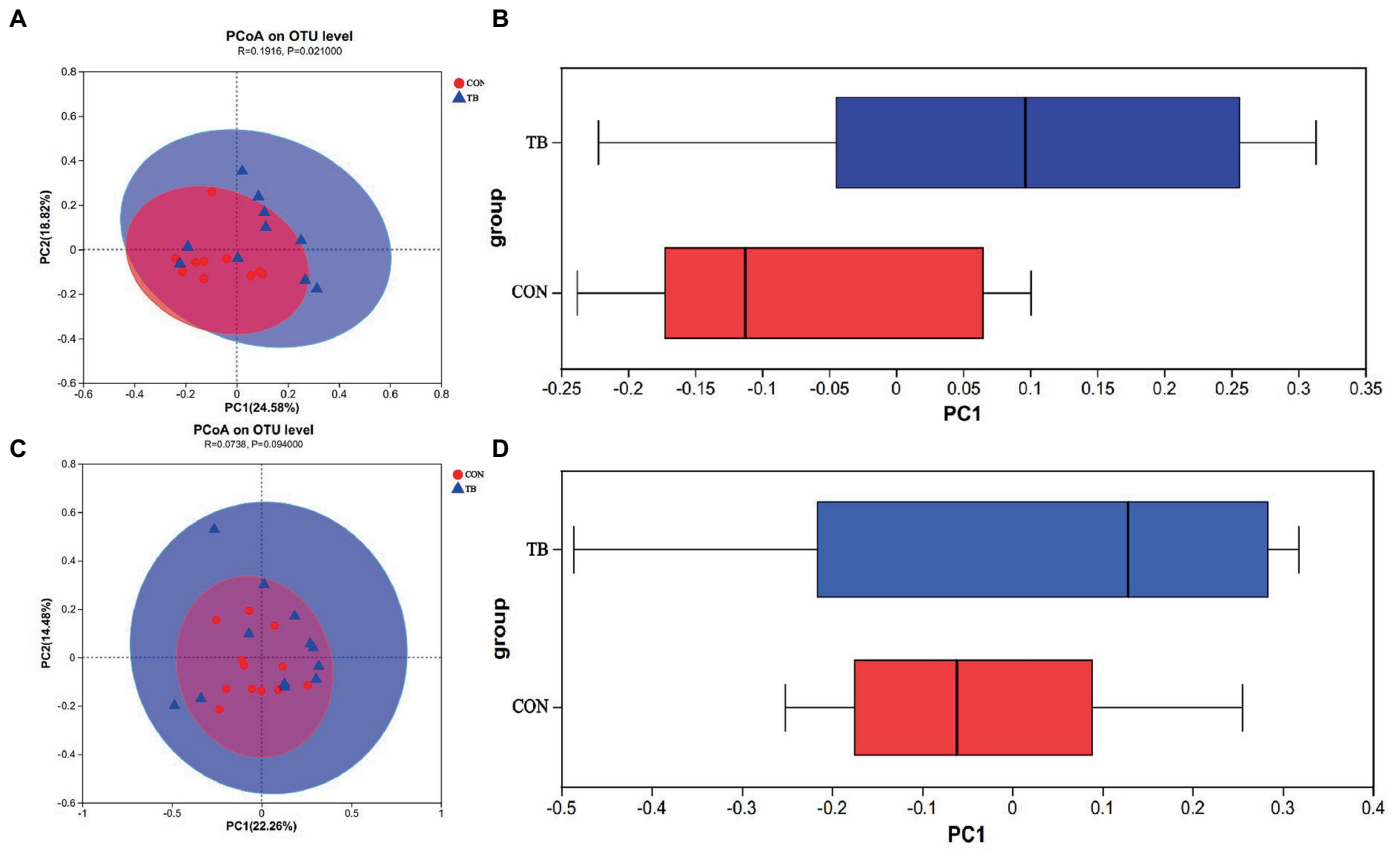
Effects of tributyrin on the fecal microbes of sows **(A,B)** and piglets **(C,D)**. Principal coordinate analysis (PCoA) plots show differences between the CON and TB groups. Red circles indicate the CON group, blue circles the TB group, and the closer the two samples are, the more similar the composition of the two species **(A,C)**. Boxplots represent the distribution of different groups of samples on the PC1 axis. **(B,D)** *R*-value is scaled to lie between −1 and +l. Generally, 0 < *R* < 1 and *p* < 0.05 represents that there were significant differences between the groups.

Principal coordinate analysis (PCoA) revealed a significant increase in gut microbiota diversity following dietary tributyrin supplementation in sows (*p* = 0.020), and there had a tendency to increase in piglets (*p* = 0.094), compared to the CON group. As shown in Figure 4, the microbial profile of the CON group was closely clustered, indicating that their bacterial community structures were highly similar and stable, whereas the TB group showed a significant shift in the microbial community. The TB group showed a significantly improved diversity of intestinal microbes in piglets, which helped establish and improve the intestinal flora.

**Figure 5:**
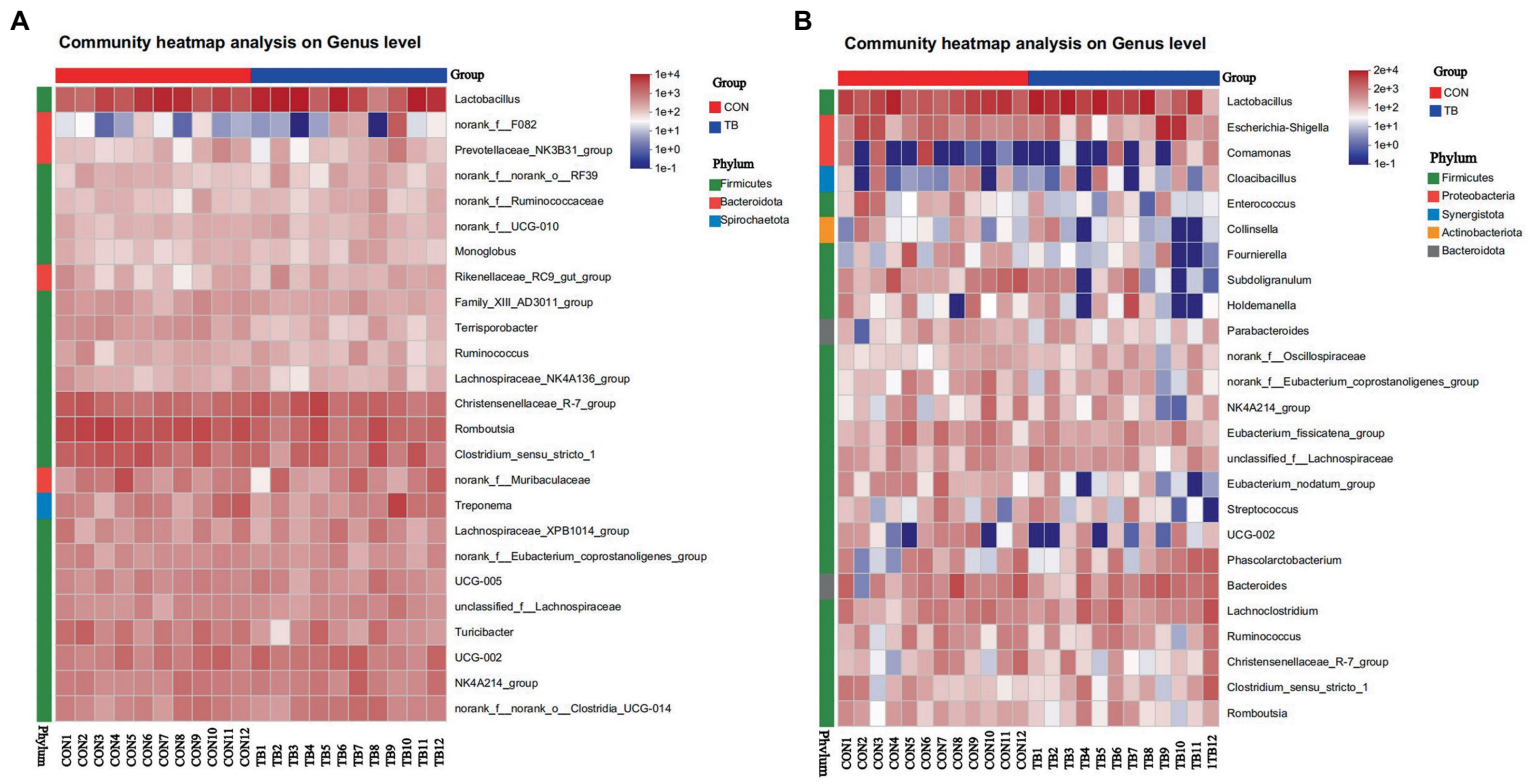
Bacterial community heatmap analysis at genus level in sows **(A)** and piglets **(B)**. CON (1–12), basal diet; TB (1–12), basal diet +500 g/t tributyrin. The abscissa is the sample name, and the ordinate is the species name. The abundance change of different species in the sample is displayed by the color gradient. The color gradient is shown on the right side of the figure.

The heatmap (Figure 5) exhibits the abundance of the selected genera across all the samples. The heatmap diagram of the sow shows 25 genera from three bacterial phyla, and 25 genera from the heatmap diagram, from five bacterial phyla. As shown in Figure 5, The results showed that the dominant genera of the piglets and sows, with no significant difference in groups.

**Figure 6:**
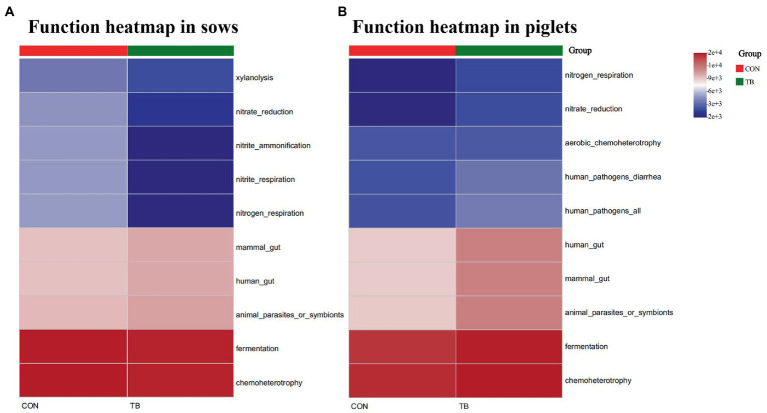
Relative abundance of the predicted function of fecal microbiota in sows **(A)** and piglets **(B)**. CON, basal diet; TB, basal diet +500 g/t tributyrin. The abscissa is the sample name, and the ordinate is the species name. The abundance change of different species in the sample is displayed by the color gradient. The color gradient is shown on the right side of the figure.

The relative abundance of top 10 predicted function of fecal microbiota in sows and piglets is shown in Supplementary Sheet 1 (sows) and Supplementary Sheet 2 (piglets), and displayed with heatmap in Figure 6. Microbial functions were predicted using FAPROTAX based on the relative abundance of fecal microbes. Among the top 10 functions, “chemoheterotrophic” and “fermentation” are the top two most important functional annotations in sows and piglets. The TB group increased the relative abundance of “Mammal_gut,” “Human_gut,” “Animal_parasites_or_symbionts” and decreased the relative abundance of “Nitrate_reduction,” “Nitrite_ammonification,” “Nitrite_respiration,” and “Nitrogen-respiration” in sows. The TB group increased the relative abundance of “Human_gut,” “Mammal_gut,” “Animal_parasites_or_symbionts,” “Chemoheterotrophy,” and “Fermentation” in piglets.

## 4. Discussion

Previous studies found that there was no significant effect on the litter performance of sows by supplementation of 1% butyrate in the diet during pregnancy (Huang et al., 2017; Chen et al., 2019). As a prodrug of butyrate, tributyrin has the same effect as butyrate (Chen et al., 2018). Similarly, maternal tributyrin supplementation had no significant effect on litter performance of sows in this experiment, which is consistent with the above research. The duration of delivery was closely related to the sow’s health and the piglet’s growth performance (Langendijk and Plush, 2019). Prolonged labor farrowing duration may damage uterine health and fertility of sows after weaning (Peltoniemi et al., 2016; Björkman et al., 2018), and increase the mortality of piglets (Gourley et al., 2020a,b; Langendijk and Plush, 2019; Van den Bosch et al., 2022). Metabolic disorders and inflammation in sows in late pregnancy, resulting in prolonged labor processes (Muns et al., 2016; Royer et al., 2016), influencing the rate of live births (Wang et al., 2019a,b; Wang H. et al., 2019), and tributyrin modulates inflammatory cytokines (Gu et al., 2017) and reduces the occurrence of oxidative stress (Wang et al., 2019a,b; Wang H. et al., 2019). After the addition of tributyrin, the levels of the anti-inflammatory cytokines IL-6 and IL-10 in sows increased, which is consistent with the above conclusions. This shows that tributyrin can reduce the occurrence of oxidative stress, shorten the labor process, and improve the reproductive performance of sows by increasing the levels of anti-inflammatory cytokines in the body.

This study showed that the addition of tributyrin increased the weaning weight of nursing piglets and significantly reduced their rate of occurrence of diarrhea. Breast milk contains many nutrients that are essential for growth and immune protection in newborn piglets (Devillers et al., 2011). The addition of tributyrin could change the composition of the milk of the sow, increasing the content of milk fat and protein in the sow’s emulsion, thereby improving the litter weight of the weaning piglets. Meanwhile, the addition of tributyrin increased the blood content of the anti-inflammatory factors IL-10, IL-6, and IgA in piglets. Previous studies have shown that IgA transfers from the breast to colostrum (Salmon et al., 2009). The cytokines present in the colostrum/milk of the sows can be transferred to the newborn piglets through breast milk, and thus the increased serum cytokine concentration in piglets may be related to the increased cytokine content in the blood of sows. The present study showed that tributyrin reduced the diarrhea rate of lactating piglets, which is consistent with previous findings (Wang et al., 2019b). After the addition of tributyrin, the levels of anti-inflammatory factors (IL-6 and IL-10) in piglets increase, which inhibits the production of proinflammatory cytokines (Opal and DePalo, 2000; Yoo and Morrison, 2005). Meanwhile, IgA obtained through colostrum also increased the piglets’ resistance and reduced the occurrence of diarrhea. Tributyrin can improve immunity and reduce diarrhea in piglets via maternal nutritional programming.

The addition of tributyrin effectively regulated the structure of the intestinal microbial flora and promoted the digestion and absorption of nutrients. Gut microbiota play a very important role in individual growth and development, and this study investigated the effects of tributyrin supplementation on the composition of intestinal microbes of not only the mother, but also the offspring. Gut microbes are related to the reproductive performance of sows (Uryu et al., 2020), and the composition of the intestinal flora affects stillbirth rate, farrowing duration, and oxidative stress state (Wang et al., 2019a,b; Wang H. et al., 2019). The results of the present study showed that the addition of tributyrin increased the relative abundance of Bacteroidota and Spirochaetota in the intestines of sows at the phylum level, and the relative abundance of Lactobacillales, Lactobacillaceae, and Lactobacillus in the intestines of piglets and sows also increased. Firmicutes are known for their ability to produce butyrate, whereas Bacteroidetes have acetate and propionate as their main metabolites (Ríos-Covián et al., 2016). Short-chain fatty acids are important for maintaining the health of the hosts and sows, after the addition of TB, the increasing abundance of microbiota producing SCFA and beneficial bacteria (e.g., Lactobacillus) can also help improve gut health and immunity (Huang et al., 2004). Maternal gut microbes colonize the fetus through the uterus during pregnancy (Collado et al., 2016; Rackaityte et al., 2020). Furthermore, the microbiota in the mother can be transmitted to the offspring after delivery through direct contact with the offspring or by breastfeeding during lactation (Pannaraj et al., 2017; Wampach et al., 2018). Thus, it can be speculated that after the addition of TB, the gut microbiota of sows is changed and simultaneously transmitted to the offspring. The increase of Lactobacillus can effectively improve the immune system and reduce the rate of diarrhea in piglets (Yang et al., 2015). Furthermore, nutrient digestibility of feed is closely related to the intestinal health of animals. Tributyrin promotes intestinal development by supplying energy and maintaining the balance of bacterial flora (Gaschott et al., 2001). The present study found that the addition of 0.05% tributyrin increased the GE and fat digestibility of lactating sow. This shows that tributyrin supplementation can effectively regulate the intestinal microbial community to affect nutrient digestibility, thus greatly influencing the growth and development of animals (Lu et al., 2021). In our study, the TB group decreased predicted functions“Nitrate_respiration,” which is related to aerobic respiration, were observed in the sows. The results suggested that tributyrin supplementation could promote the proliferation of beneficial bacteria and inhibit the proliferation of harmful bacteria (Li et al., 2020).

## 5. Conclusion

In conclusion, tributyrin supplementation shortened the farrowing duration and reduced the diarrhea rate of piglets by improving the inflammatory response and composition of gut microbiota in piglets and sows. This research provides theoretical support for further application of tributyrin in improving reproductive performance and promoting animal gut health.

## Data availability statement

The datasets presented in this study can be found in online repositories. The names of the repository/repositories and accession number(s) can be found in the article/Supplementary material.

## Ethics statement

The animal study was reviewed and approved by the Institutional Animal Care and Use Committee of Sichuan Agricultural University (SCAUAC202108-3).

## Author contributions

YL and ZM conceived and designed the study. YL, YW, ZM, LC, ZF, and SX performed the experiments and analyzed the data. BF, JL, LH, JZ, DW, and YZ performed the lab analysis. YL and DL wrote and edited the original draft. All authors have read and agreed to the published version of the manuscript.

## Funding

This study was funded by the National Key R&D Program of China (2021YFD1300202), the Sichuan Major Science and Technology Projects (2021ZDZX0009), and the Perstorp (Shanghai) Chemical Trading Co., Ltd. (Shanghai, China).

## Conflict of interest

YW was employed by the company Perstorp (Shanghai) Chemical Trading Co., Ltd.

The remaining authors declare that the research was conducted in the absence of any commercial or financial relationships that could be construed as a potential conflict of interest.

## Publisher’s note

All claims expressed in this article are solely those of the authors and do not necessarily represent those of their affiliated organizations, or those of the publisher, the editors and the reviewers. Any product that may be evaluated in this article, or claim that may be made by its manufacturer, is not guaranteed or endorsed by the publisher.

## References

[ref9001] AOAC (2012). Official Methods of Analysis. 19th Edn, Association of Official Analytical Chemists, Arlington, VA.

[ref1] Augustin M. A. Abeywardena M. Y. Patten G. Head R. Lockett T. De Luca A.. (2011). Effects of microencapsulation on the gastrointestinal transit and tissue distribution of a bioactive mixture of fish oil, tributyrin and resveratrol. J. Funct. Foods 3, 25–37. doi: 10.1016/j.jff.2011.01.003

[ref9002] Biagi G. Piva A. Moschini M. Vezzali E. Roth F. X. (2007). Performance, intestinal microflora, and wall morphology of weanling pigs fed sodium butyrate. J. Anim. Sci. 85, 1184–1191. doi: 10.2527/jas.2006-378 17296766

[ref2] Björkman S. Oliviero C. Kauffold J. Soede N. M. Peltoniemi O. (2018). Prolonged parturition and impaired placenta expulsion increase the risk of postpartum metritis and delay uterine involution in sows. Theriogenology 106, 87–92. doi: 10.1016/j.theriogenology.2017.10.003, PMID: 29040880

[ref3] Chen J. Xu Q. Li Y. Tang Z. Sun W. Zhang X.. (2019). Comparative effects of dietary supplementations with sodium butyrate, medium-chain fatty acids, and n-3 polyunsaturated fatty acids in late pregnancy and lactation on the reproductive performance of sows and growth performance of suckling piglets. J. Anim. Sci. 97, 4256–4267. doi: 10.1093/jas/skz284, PMID: 31504586 PMC6776281

[ref4] Chen S. Zhou Y. Chen Y. Gu J. (2018). fastp: An ultra-fast all-in-one FASTQ preprocessor. Bioinformatics 34, i884–i890. doi: 10.1093/bioinformatics/bty560, PMID: 30423086 PMC6129281

[ref5] Collado M. C. Rautava S. Aakko J. Isolauri E. Salminen S. (2016). Human gut colonisation may be initiated *in utero* by distinct microbial communities in the placenta and amniotic fluid. Sci. Rep. 6:23129. doi: 10.1038/srep23129, PMID: 27001291 PMC4802384

[ref6] Devillers N. Le Dividich J. Prunier A. (2011). Influence of colostrum intake on piglet survival and immunity. Animal 5, 1605–1612. doi: 10.1017/S175173111100067X, PMID: 22440352

[ref7] Dong L. Zhong X. He J. Zhang L. Bai K. Xu W.. (2016). Supplementation of tributyrin improves the growth and intestinal digestive and barrier functions in intrauterine growth-restricted piglets. Clin. Nutr. 35, 399–407. doi: 10.1016/j.clnu.2015.03.002, PMID: 26112894

[ref9] Gaschott T. Steinhilber D. Milovic V. Stein J. (2001). Tributyrin, a stable and rapidly absorbed prodrug of butyric acid, enhances antiproliferative effects of dihydroxycholecalciferol in human colon cancer cells. J. Nutr. 131, 1839–1843. doi: 10.1093/jn/131.6.1839, PMID: 11385076

[ref10] Gong L. Xiao G. Zheng L. Yan X. Qi Q. Zhu C.. (2021). Effects of dietary tributyrin on growth performance, biochemical indices, and intestinal microbiota of yellow-feathered broilers. Animals 11:3425. doi: 10.3390/ani11123425, PMID: 34944202 PMC8697914

[ref11] Gourley K. M. Calderon H. I. Woodworth J. C. DeRouchey J. M. Tokach M. D. Dritz S. S.. (2020a). Sow and piglet traits associated with piglet survival at birth and to weaning. J. Anim. Sci. 98:skaa 187. doi: 10.1093/jas/skaa187, PMID: 32506128 PMC7311083

[ref12] Gourley K. M. Swanson A. J. Royall R. Q. DeRouchey J. M. Tokach M. D. Dritz S. S.. (2020b). Effects of timing and size of meals prior to farrowing on sow and litter performance. Transl. Anim. Sci. 4, 724–736. doi: 10.1093/tas/txaa066, PMID: 32705061 PMC7281871

[ref13] Gu Y. Song Y. Yin H. Lin S. Zhang X. Che L.. (2017). Dietary supplementation with tributyrin prevented weaned pigs from growth retardation and lethal infection via modulation of inflammatory cytokines production, ileal expression, and intestinal acetate fermentation. J. Anim. Sci. 95, 226–238. doi: 10.2527/jas.2016.0911, PMID: 28177354

[ref14] He B. Wang M. Guo H. Jia Y. Yang X. Zhao R. (2016). Effects of sodium butyrate supplementation on reproductive performance and colostrum composition in gilts. Animal 10, 1722–1727. doi: 10.1017/S1751731116000537, PMID: 27040131

[ref9003] Huang Y. Gao S. Jun G. Zhao R. Yang X. (2017). Supplementing the maternal diet of rats with butyrate enhances mitochondrial biogenesis in the skeletal muscles of weaned offspring. Br. J. Nutr. 117, 12–20. doi: 10.1017/S0007114516004402 28091351

[ref17] Huang C. Qiao S. Li D. Piao X. Ren J. (2004). Effects of lactobacilli on the performance, diarrhea incidence, VFA concentration and gastrointestinal microbial flora of weaning pigs. J. Anim. Sci. 17, 401–409. doi: 10.5713/ajas.2004.401

[ref21] Langendijk P. Plush K. (2019). Parturition and its relationship with stillbirths and asphyxiated piglets. Animals 9:885. doi: 10.3390/ani9110885, PMID: 31683527 PMC6912372

[ref22] Li Y. Zhao X. Zhang L. Zhan X. Liu Z. Zhuo Y.. (2020). Effects of a diet supplemented with exogenous catalase from *Penicillium notatum* on intestinal development and microbiota in weaned piglets. Microorganisms 8:391. doi: 10.3390/microorganisms8030391, PMID: 32168962 PMC7143822

[ref23] Long S. Hu J. Mahfuz S. Ma H. Piao X. (2021). Effects of dietary supplementation of compound enzymes on performance, nutrient digestibility, serum antioxidant status, immunoglobulins, intestinal morphology and microbiota community in weaned pigs. Arch. Anim. Nutr. 75, 31–47. doi: 10.1080/1745039X.2020.1852008, PMID: 33317350

[ref24] Lu C. Liu Y. Ma Y. Wang S. Cai C. Yang Y.. (2021). Comparative evaluation of the ileum microbiota composition in piglets at different growth stages. Front. Microbiol. 12:765691. doi: 10.3389/fmicb.2021.765691, PMID: 34925272 PMC8672721

[ref25] Lu H. Su S. Ajuwon K. M. (2012). Butyrate supplementation to gestating sows and piglets induces muscle and adipose tissue oxidative genes and improves growth performance. J. Anim. Sci. 90, 430–432. doi: 10.2527/jas.53817, PMID: 23365400

[ref9004] Mallo J. J. Balfagón A. Gracia M. I. Honrubia P. Puyalto M. (2012). Evaluation of different protections of butyric acid aiming for release in the last part of the gastrointestinal tract of piglets. J. Anim. Sci. 90, 227–229. doi: 10.2527/jas.53959 23365338

[ref27] Miragoli F. Patrone V. Prandini A. Sigolo S. Dell’Anno M. Rossi L.. (2021). Implications of Tributyrin on gut microbiota shifts related to performances of weaning piglets. Microorganisms 9:584. doi: 10.3390/microorganisms9030584, PMID: 33809105 PMC8001585

[ref28] Miyoshi M. Sakaki H. Usami M. Iizuka N. Shuno K. Aoyama M.. (2011). Oral administration of tributyrin increases concentration of butyrate in the portal vein and prevents lipopolysaccharide-induced liver injury in rats. Clin. Nutr. 30, 252–258. doi: 10.1016/j.clnu.2010.09.012, PMID: 21051124

[ref29] Moquet P. C. A. Onrust L. van Immerseel F. Ducatelle R. Hendriks W. H. Kwakkel R. P. (2016). Importance of release location on the mode of action of butyrate derivatives inthe avian gastrointestinal tract. Worlds Poult. Sci. J. 72, 61–80. doi: 10.1017/S004393391500269X

[ref9005] Muns R. Malmkvist J. Larsen M. L. Sørensen D. Pedersen L. J. (2016). High environmental temperature around farrowing induced heat stress in crated sows. J. Anim. Sci. 94, 377–384. doi: 10.2527/jas.2015-9623 26812342

[ref9006] NRC. (2012). Nutrient Requirements of Swine, 11th ed. The National Academies Press: Washington, DC, USA.

[ref30] Oliviero C. Heinonen M. Valros A. Peltoniemi O. (2010). Environmental and sow-related factors affecting the duration of farrowing. Anim. Reprod. Sci. 119, 85–91. doi: 10.1016/j.anireprosci.2009.12.009, PMID: 20053511

[ref31] Opal S. M. DePalo V. A. (2000). Anti-inflammatory cytokines. Chest 117, 1162–1172. doi: 10.1378/chest.117.4.1162 10767254

[ref32] Pannaraj P. S. Li F. Cerini C. Bender J. M. Yang S. Rollie A.. (2017). Association between breast milk bacterial communities and establishment and development of the infant gut microbiome. JAMA Pediatr. 171, 647–654. doi: 10.1001/jamapediatrics.2017.0378, PMID: 28492938 PMC5710346

[ref35] Peltoniemi O. Björkman S. Oliviero C. (2016). Parturition effects on reproductive health in the gilt and sow. Reprod. Domest. Anim. 51, 36–47. doi: 10.1111/rda.12798, PMID: 27762056

[ref36] Rackaityte E. Halkias J. Fukui E. M. Mendoza V. F. Hayzelden C. Crawford E. D.. (2020). Viable bacterial colonization is highly limited in the human intestine *in utero*. Nat. Med. 26, 599–607. doi: 10.1038/s41591-020-0761-3 32094926 PMC8110246

[ref37] Ríos-Covián D. Ruas-Madiedo P. Margolles A. Gueimonde M. de Los Reyes-Gavilán C. G. Salazar N. (2016). Intestinal short chain fatty acids and their link with diet and human health. Front. Microbiol. 7:185. doi: 10.3389/fmicb.2016.00185, PMID: 26925050 PMC4756104

[ref9007] Royer E. Barbé F. Guillou D. Rousselière Y. Chevaux E. (2016). Development of an oxidative stress model in weaned pigs highlighting plasma biomarkers’ specificity to stress inducers.J. Anim. Sci. 94, 48–53. doi: 10.2527/jas.2015-9857

[ref38] Salmon H. Berri M. Gerdts V. Meurens F. (2009). Humoral and cellular factors of maternal immunity in swine. Dev. Comp. Immunol. 33, 384–393. doi: 10.1016/j.dci.2008.07.007, PMID: 18761034

[ref39] Siddiqui M. T. Cresci G. A. M. (2021). The Immunomodulatory functions of butyrate. J. Inflamm. Res. 14, 6025–6041. doi: 10.2147/JIR.S300989, PMID: 34819742 PMC8608412

[ref40] Sotira S. Dell’Anno M. Caprarulo V. Hejna M. Pirrone F. Callegari M. L.. (2020). Effects of tributyrin supplementation on growth performance, insulin, blood metabolites and gut microbiota in weaned piglets. Animals 10:726. doi: 10.3390/ani10040726, PMID: 32331306 PMC7222802

[ref41] Uryu H. Tsukahara T. Ishikawa H. Oi M. Otake S. Yamane I.. (2020). Comparison of productivity and fecal microbiotas of sows in commercial farms. Microorganisms 8:1469. doi: 10.3390/microorganisms8101469, PMID: 32987859 PMC7599717

[ref42] Van den Bosch M. van de Linde I. B. Kemp B. van den Brand H. (2022). Disentangling litter size and farrowing duration effects on piglet stillbirth, acid-base blood parameters and pre-weaning mortality. Front. Vet. Sci. 9:836202. doi: 10.3389/fvets.2022.836202, PMID: 35529832 PMC9071363

[ref43] Wampach L. Heintz-Buschart A. Fritz J. V. Ramiro-Garcia J. Habier J. Herold M.. (2018). Birth mode is associated with earliest strain-conferred gut microbiome functions and immunostimulatory potential. Nat. Commun. 9:5091. Published 2018 Nov 30. doi: 10.1038/s41467-018-07631-x, PMID: 30504906 PMC6269548

[ref44] Wang C. Cao S. Shen Z. Hong Q. Feng J. Peng Y.. (2019a). Effects of dietary tributyrin on intestinal mucosa development, mitochondrial function and AMPK-mTOR pathway in weaned pigs. J. Anim. Sci Biotechnol. 10:93. doi: 10.1186/s40104-019-0394-x, PMID: 31788241 PMC6876078

[ref45] Wang Q. Garrity G. M. Tiedje J. M. Cole J. R. (2007). Naive Bayesian classifier for rapid assignment of rRNA sequences into the new bacterial taxonomy. Appl. Environ. Microbiol. 73, 5261–5267. doi: 10.1128/AEM.00062-07, PMID: 17586664 PMC1950982

[ref46] Wang H. Hu C. Cheng C. Cui J. Ji Y. Hao X.. (2019). Unraveling the association of fecal microbiota and oxidative stress with stillbirth rate of sows. Theriogenology 136, 131–137. doi: 10.1016/j.theriogenology.2019.06.028, PMID: 31255919

[ref48] Wang C. Shen Z. Cao S. Zhang Q. Peng Y. Hong Q.. (2019b). Effects of tributyrin on growth performance, intestinal microflora and barrier function of weaned pigs. Anim. Feed Sci. Technol. 258:114311. doi: 10.1016/j.anifeedsci.2019.114311

[ref49] Wang Y. Wang Y. Lin X. Gou Z. Fan Q. Jiang S. (2021). Effects of clostridium butyricum, sodium butyrate, and butyric acid glycerides on the reproductive performance, egg quality, intestinal health, and offspring performance of yellow-feathered breeder hens. Front. Microbiol. 12:657542. doi: 10.3389/fmicb.2021.657542, PMID: 34603221 PMC8481923

[ref50] Wang J. Zhang H. Bai S. Zeng Q. Su Z. Zhuo Y.. (2021). Dietary tributyrin improves reproductive performance, antioxidant capacity, and ovary function of broiler breeders. Poult. Sci. 100:101429. doi: 10.1016/j.psj.2021.101429, PMID: 34555757 PMC8458981

[ref52] Yang F. Hou C. Zeng X. Qiao S. (2015). The use of lactic acid bacteria as a probiotic in swine diets. Pathogens 4, 34–45. doi: 10.3390/pathogens4010034, PMID: 25633489 PMC4384071

[ref54] Yoo E. M. Morrison S. L. (2005). IgA: An immune glycoprotein. Clin. Immunol. 116, 3–10. doi: 10.1016/j.clim.2005.03.010 15925826

