# Supplementary material for: Maternal tributyrin supplementation in late pregnancy and lactation improves offspring immunity, gut microbiota, and diarrhea rate in a sow model
Source: Front Microbiol. 2023 Apr 12;14:1142174. doi: 10.3389/fmicb.2023.1142174 (PMC10165498; doi:10.3389/fmicb.2023.1142174)
Supplement: Supplementary file 1 [file Table_1.docx]

Supplementary Material

## **Supplementary Table 1.** The nutritional parameters of the gestation and lactation diet

| **Items** | **Gestation** | **Lactation** |
| --- | --- | --- |
| Net Energy, kcal/kg | 2300 | 2650 |
| Crude Protein, (%) | 13% | 16% |
| Crude Fat, (%) | 4% | 5.55% |
| Total Lysine, (%) | 0.75 | 1.15 |
| Ca, (%) | 0.70 | 0.90 |
| P, (%) | 0.60 | 0.65 |
